# Author Correction: The role of energy storage in deep decarbonization of electricity production

**DOI:** 10.1038/s41467-019-11778-6

**Published:** 2019-08-26

**Authors:** Maryam Arbabzadeh, Ramteen Sioshansi, Jeremiah X. Johnson, Gregory A. Keoleian

**Affiliations:** 1 Center for Sustainable Systems, School for Environment and Sustainability, University of Michigan, Ann Arbor, 48109 MI USA; 2 Department of Integrated Systems Engineering, The Ohio State University, Columbus, 43210 OH USA; 3 Department of Civil, Construction, and Environmental Engineering, North Carolina State University, Raleigh, 27607 NC USA

**Keywords:** Energy and society, Environmental sciences, Energy science and technology

Correction to: *Nature Communications* 10.1038/s41467-019-11161-5, published online 30 July 2019.

The original version of this Article contained an error in Figs. 3 and 4, in which all *y*-axis labels incorrectly included ‘Emissions reduction (million ton)’ rather than the correct ‘Total emissions (million ton)’. This has been corrected in both the PDF and HTML versions of the Article.

