# Supplementary material for: The role of energy storage in deep decarbonization of electricity production
Source: Nat Commun. 2019 Jul 30;10:3413. doi: 10.1038/s41467-019-11161-5 (PMC6667472; doi:10.1038/s41467-019-11161-5)
Supplement: Supplementary file 1 — Supplementary Information [file 41467_2019_11161_MOESM1_ESM.pdf]

Supplementary Information

The role of energy storage in deep decarbonization of electricity production

Arbabzadeh *et al*.

Supplementary Table 1: Energy storage capacity (GWh) installed in the CAISO system in 2012 with different wind- and solar-penetration levels and CO_2-emissions taxes in the base case with a 7.0-GW minimum-dispatchability requirement.

Technology	Solar	\$0 per ton Tax			\$50 per ton Tax			\$100 per ton Tax			\$200 per ton Tax		
	(GW)	0 GW Wind	10 GW Wind	20 GW Wind	0 GW Wind	10 GW Wind	20 GW Wind	0 GW Wind	10 GW Wind	20 GW Wind	0 GW Wind	10 GW Wind	20 GW Wind
PHS	0	0.0	0.0	0.0	0.0	0.0	0.0	0.0	0.0	0.0	0.0	0.0	7.5
	20	0.0	0.0	0.0	0.0	0.0	15.7	0.1	7.8	50.0	7.2	16.3	120.3
	40	27.7	33.0	37.2	60.9	67.4	97.4	72.8	84.7	155.9	85.1	172.2	190.1
ACAES	0	0.0	0.0	0.0	0.0	0.0	0.0	0.0	0.0	0.0	0.0	0.0	0.0
	20	0.0	0.0	0.0	0.0	0.0	0.0	0.0	0.0	1.3	2.4	7.3	13.8
	40	0.0	0.0	0.0	30.1	33.2	25.4	52.5	57.1	47.1	70.6	76.1	69.9
DCAES	0	0.0	0.0	0.0	0.0	0.0	0.0	0.0	0.0	0.0	0.0	0.0	12.0
	20	0.0	0.0	0.0	0.0	0.0	17.6	0.0	8.6	71.4	10.9	31.3	207.7
	40	36.3	34.8	24.9	91.2	100.9	109.6	116.6	137.7	143.9	167.1	217.8	236.1
PbA	0	0.0	0.0	0.0	0.0	0.0	0.0	0.0	0.0	0.0	0.0	0.0	0.0
	20	0.0	0.0	0.0	0.0	0.0	0.0	0.0	0.0	0.0	0.0	0.0	0.0
	40	0.0	0.0	0.0	0.0	0.0	0.0	0.0	0.0	0.0	33.9	36.5	28.6
VRB	0	0.0	0.0	0.0	0.0	0.0	0.0	0.0	0.0	0.0	0.0	0.0	0.0
	20	0.0	0.0	0.0	0.0	0.0	0.0	0.0	0.0	0.0	0.0	0.0	0.0
	40	0.0	0.0	0.0	0.0	0.0	0.0	6.1	2.0	0.0	42.8	45.7	36.2
NaS	0	0.0	0.0	0.0	0.0	0.0	0.0	0.0	0.0	0.0	0.0	0.0	0.0
	20	0.0	0.0	0.0	0.0	0.0	0.0	0.0	0.0	0.0	0.0	0.0	0.0
	40	0.0	0.0	0.0	0.0	0.0	0.0	2.8	0.0	0.0	37.3	39.9	32.5
PSB	0	0.0	0.0	0.0	0.0	0.0	0.0	0.0	0.0	0.0	0.0	0.0	0.0
	20	0.0	0.0	0.0	0.0	0.0	0.0	0.0	0.0	0.0	0.0	0.0	0.9
	40	0.0	0.0	0.0	0.0	0.0	0.0	18.3	19.0	12.7	43.5	48.6	41.2
ZNBR	0	0.0	0.0	0.0	0.0	0.0	0.0	0.0	0.0	0.0	0.0	0.0	0.0
	20	0.0	0.0	0.0	0.0	0.0	0.0	0.0	0.0	0.0	0.0	0.0	0.0
	40	0.0	0.0	0.0	0.0	0.0	0.0	0.0	0.0	0.0	28.4	31.8	26.9

Supplementary Table 2: Energy storage capacity (GWh) installed in the ERCOT system in 2012 with different wind- and solar-penetration levels and CO_2-emissions taxes in the base case with an 8.2-GW minimum-dispatchability requirement.

Technology	Solar (GW)	$0 per ton Tax 0 GW Wind	$0 per ton Tax 10 GW Wind	$0 per ton Tax 20 GW Wind	$50 per ton Tax 0 GW Wind	$50 per ton Tax 10 GW Wind	$50 per ton Tax 20 GW Wind	$100 per ton Tax 0 GW Wind	$100 per ton Tax 10 GW Wind	$100 per ton Tax 20 GW Wind	$200 per ton Tax 0 GW Wind	$200 per ton Tax 10 GW Wind	$200 per ton Tax 20 GW Wind
PHS	0	0.0	0.0	0.0	0.0	0.0	0.0	0.0	0.0	11.0	1.9	19.8	79.7
	20	0.0	0.0	0.0	0.0	0.0	0.0	0.0	0.0	1.4	0.0	8.3	46.0
	40	0.0	0.0	0.0	0.0	4.8	18.8	10.6	22.0	45.0	37.5	56.7	83.7
ACAES	0	0.0	0.0	0.0	0.0	0.0	0.0	0.0	0.0	0.0	1.2	10.0	24.8
	20	0.0	0.0	0.0	0.0	0.0	0.0	0.0	0.0	0.0	0.0	1.6	8.4
	40	0.0	0.0	0.0	0.0	0.0	1.3	1.9	7.3	12.6	23.2	21.9	25.0
DCAES	0	0.0	0.0	0.0	0.0	0.0	0.0	9.1	28.3	86.2	103.1	122.7	249.3
	20	0.0	0.0	0.0	0.0	0.0	0.0	0.0	0.0	33.5	75.1	97.8	149.7
	40	0.0	0.0	0.0	4.1	15.6	42.8	51.6	59.8	89.0	159.1	151.4	155.5
PbA	0	0.0	0.0	0.0	0.0	0.0	0.0	0.0	0.0	0.0	0.0	0.0	0.0
	20	0.0	0.0	0.0	0.0	0.0	0.0	0.0	0.0	0.0	0.0	0.0	0.0
	40	0.0	0.0	0.0	0.0	0.0	0.0	0.0	0.0	0.8	0.6	3.5	5.3
VRB	0	0.0	0.0	0.0	0.0	0.0	0.0	0.0	0.0	0.0	0.0	0.0	0.0
	20	0.0	0.0	0.0	0.0	0.0	0.0	0.0	0.0	0.0	0.0	0.0	0.0
	40	0.0	0.0	0.0	0.0	0.0	0.0	0.0	0.0	0.5	3.4	6.1	9.7
NaS	0	0.0	0.0	0.0	0.0	0.0	0.0	0.0	0.0	0.0	0.0	0.0	0.0
	20	0.0	0.0	0.0	0.0	0.0	0.0	0.0	0.0	0.0	0.0	0.0	0.0
	40	0.0	0.0	0.0	0.0	0.0	0.0	0.0	0.0	0.7	1.3	4.3	7.0
PSB	0	0.0	0.0	0.0	0.0	0.0	0.0	0.0	0.0	0.0	0.0	0.0	0.0
	20	0.0	0.0	0.0	0.0	0.0	0.0	0.0	0.0	0.0	0.0	0.0	0.0
	40	0.0	0.0	0.0	0.0	0.0	0.0	0.0	0.0	1.5	2.1	5.1	9.4
ZNBR	0	0.0	0.0	0.0	0.0	0.0	0.0	0.0	0.0	0.0	0.0	0.0	0.0
	20	0.0	0.0	0.0	0.0	0.0	0.0	0.0	0.0	0.0	0.0	0.0	0.0
	40	0.0	0.0	0.0	0.0	0.0	0.0	0.0	0.0	0.5	0.0	0.9	2.9
