# Supplementary material for: The role of energy storage in deep decarbonization of electricity production
Source: Nat Commun. 2019 Jul 30;10:3413. doi: 10.1038/s41467-019-11161-5 (PMC6667472; doi:10.1038/s41467-019-11161-5)
Supplement: Supplementary file 3 — Description of Additional Supplementary Files [file 41467_2019_11161_MOESM3_ESM.pdf]

Description of Additional Supplementary Files

File Name: Supplementary Data 1

Description: Supplementary Data 1 summarizes the amounts of installed energy storage, renewable curtailment, and CO_2 emissions in the other years and with the other minimum-dispatchability requirements that we analyze.
